# Surface Integrity and Tool Wear Analysis on Turning of Copper-Nickel 70/30 ASTM B122 Alloy under Low Initial Lubrication

**DOI:** 10.3390/ma14174868

**Published:** 2021-08-27

**Authors:** Enrique García-Martínez, Valentín Miguel, Alberto Martínez-Martínez, María Carmen Manjabacas, Juana Coello

**Affiliations:** 1 High Technical School of Industrial Engineers of Albacete, University of Castilla—La Mancha, 02071 Albacete, Spain; Enrique.GMartinez@uclm.es (E.G.-M.); valentin.miguel@uclm.es (V.M.); juana.coello@uclm.es (J.C.); 2 Regional Development Institute, Science and Engineering of Materials, University of Castilla—La Mancha, 02071 Albacete, Spain; alberto.martinez@uclm.es

**Keywords:** Cu-Ni alloys, friction, lubrication, wear

## Abstract

Traditional flood lubrication in machining processes is considered an unsustainable technique. In this paper, the low initial lubrication (LIL) technique is analysed during turning of cupronickel 70/30 alloy, in terms of surface roughness. A tribological analysis has been developed on a pin-on-disk tribometer comparing different lubrication systems, obtaining comparative results of friction and tool wear. It has been found that the tool wear is 73% lower in comparison to flood lubrication. LIL technique shows the ability to reduce the friction coefficient compared to dry machining and leads to improve tool wear in comparison with flood lubrication. The surface integrity evaluation of machined parts finds that the LIL technique can improve the surface roughness under specific machining conditions.

## 1. Introduction

The surface after machining processes becomes essential in alloys like Cu-Ni 70/30 due to its applications [1]. If they are not manufactured correctly, corrosion phenomena can be accelerated, and the service life of the part is reduced. Thus, Gravier et al. have demonstrated that the surface roughness is highly correlated to the corrosion behavior of copper in sea water environments [2]. During the turning process of Cu-Ni 70/30 alloy, long, ductile chips are formed, which causes a high temperature rise under dry machining conditions, making some kind of lubrication necessary. However, machining processes such as turning or milling have not been widely studied on Cu-Ni 70/30 alloy, making it necessary to dig deeper into the tribological characterization of the cutting process, as well as a complete analysis of the surface roughness of the machined part. Recently, Eder et al. [3] have studied the temperature influence on the deformation behavior and subsurface alterations under dry sliding for some copper-nickel alloys. They have found that the temperature plays an important role, indicating the importance of an efficient machining process for these alloys.

It has been determined that the cutting parameters influence the surface integrity of the machined part. Altas et al. [4] reported that the feed rate and nose radius are the most determinant parameters on surface roughness when milling a nickel-titanium alloy. Mane et al. [5] also reported that the depth of cut does not influence on the control of surface roughness when turning steel. For their part, Duc et al. [6] found that the inclination angle is the major factor affecting the tool wear and the surface roughness on hard turning and that there is an optimal inclination and rake angle that provides the best cutting condition. Besides, tool wear affects the evolution of surface roughness and, in addition, friction coefficient depends on the adhesion and surface integrity of the machined part. Moreover, the cutting conditions can affect the subsurface characteristics of the machined workpiece. Anwar et al. [7] found that the cutting speed is determinant in the deformed layer, which produces a hardening effect below the machined surface. On their part, Yao et al. [8] discussed the influence of the surface integrity in the fatigue behavior of the workpiece after machining operations.

The tribological analysis allows us to know the friction phenomenon between the material of the cutting tool and the Cu-Ni alloy. The control of a low friction coefficient during the cutting process is incredibly beneficial. The increment of the friction coefficient between tool and chip leads to an excessive generation of heat in the rake face, which causes the thermal softening of the cutting tool, the shortening of the tool life and, moreover affects the surface quality and the dimensional precision. Miguélez et al. [9] also reported the influence of the friction coefficient on residual stresses by FEM simulation, being this effect variable depending on the distance from the surface. Moreover, most of the times, the lower the friction coefficient, the fewer phenomena of adhesion and dragging or plastic deformation on the machined part, as it was observed by Faverjon et al. [10] on ball-on-disk tests of aluminium. Conversely, García et al. [11] recently found that the increment of the friction coefficient is not always related to the wear increase. In fact, there are different mechanisms that influence on the wear evolution, such as chemical affinities, oxidation or changes in the mechanical properties of the contact pairs. On the other hand, Yap et al. [12] found an improvement of the surface roughness when the friction coefficient was reduced by lubrication. In any case, the phenomenon of friction in the machining process has traditionally generated great interest among the researchers, being an important factor for the process modelling. In the current practice, the Coulomb friction model is extensively used to characterize the friction coefficient between the chip and the rake face of the tool. Under this assumption, the coefficient of friction is constant in the tool-chip interface. Nevertheless, different models have been proposed that distinguish a sticking zone and a sliding zone near the tool tip [13].

Unfortunately, the measurement and control of the coefficient of friction during turning is not easy, as the speed of the cutting process and the evacuation of the chip make the measurement impossible to carry out. Some authors [14] have estimated the friction coefficient by a theoretical analysis from the measurement of cutting forces and chip morphology. However, the reliability of this procedure is sometimes questionable, as it is an indirect measurement process where several experimental errors might be added. Taking into account the previous explanations, a tribological analysis of the tool-workpiece contact in a conventional tribometer can contribute to characterize the friction phenomenon at machining speeds, as well as tool wear. Zemzemi et al. [15] developed a methodology for friction coefficient determination on high speed dry cutting. They found that the apparent friction coefficient is mainly determined by adhesive phenomena. Smolenicki et al. [16] studied the friction phenomenon during orthogonal cutting and found that the friction coefficient is a function of the cutting speed. They also reported the effect of surface oxidation during the machining process on the coefficient of friction. Mane et al. [17] measured the apparent friction coefficient between Ti6Al4V alloy and an uncoated tungsten carbide tool on an open-designed tribometer. They reported that the cutting speed showed a high influence on the friction coefficient and adhesive phenomena.

The influence of coolants and lubricants on friction coefficient under different cutting conditions have also been analysed. Thus, flood lubrication is the traditional approach to reduce friction, excessive cutting temperatures and tool wear. However, Childs [18] found that only at very low cutting speed, is flood lubrication effective in friction reduction.

Furthermore, due to the serious contamination problems that the excessive use of lubricants causes to the atmosphere, traditional flood lubrication is not considered an eco-friendly method. Moreover, the need to find more efficient and ecological methods of lubrication during machining processes has grown exponentially. Techniques such as minimal quantity of lubricant (MQL), cryogenic lubrication with liquid nitrogen, LN_2_, or laser assisted machining are being widely studied in some materials machining processes, but the efficiency of these lubrication techniques strongly depends on the machining process and the cutting conditions [19]. Although some authors have reported the beneficial effect of cryogenic lubrication on the surface roughness, the impact on the tool wear is not clear for all cutting processes. Moreover, a negative effect has been reported due to the material hardening at low temperature [20]. On the other hand, MQL has been found as a solution, achieving better results than with flood lubrication. However, MQL has a great dependence on the cutting parameters and the cutting temperature, due to the lack of an effective coolant action [21]. Moreover, these techniques require either specialized equipment or a very expensive one to be implemented in the industrial environment. Recently, some authors have concluded that cryogenic machining is only economically sustainable for some specific cutting conditions and requirements. Thus, Agrawal et al. [22] reported that cryogenic procedure is only sustainable for high cutting speed, meanwhile for low speed, carbon emissions are higher than for wet machining.

For these reasons, this paper analyzes and discusses, from a tribological viewpoint, the influence of the low initial lubrication (LIL) technique, where only a small amount of lubricant is supplied to the part at the beginning of the machining process. LIL technique is applied to the ASTM B122 70/30 alloy turning process. The effects of LIL lubrication are compared to dry and flood machining in terms of surface roughness, friction coefficient and tool wear. The approach of this paper is applicable to other different materials, and it would allow us to understand if this lubrication technique could be used in the industrial field by substituting the traditional flood lubrication. In a previous study [1], it was found that LIL technique is effective on the reduction of the cutting forces in comparison with dry machining. Besides, for finishing operations, better results were achieved than with flood lubrication, which leads to important savings in lubricants and less environmental impact.

### Analytical Fundamentals

**Figure 1:**
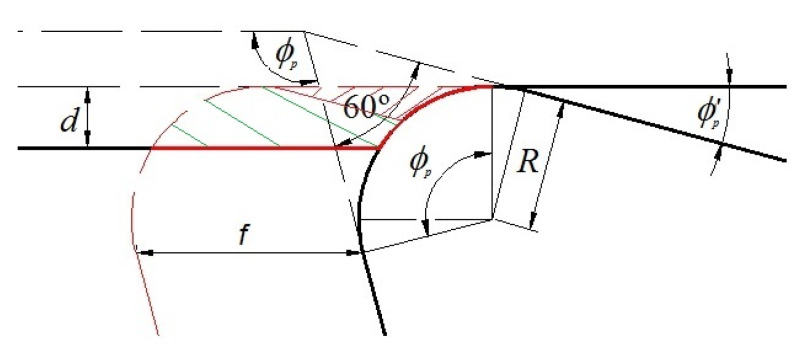
Sketch of the geometry of the turning process, initial chip section and non-machined zone for low values of feed.

During the turning process, the cutting tool moves in axial direction at a given feed rate per revolution, *f*, meanwhile the machined part rotates. The composition of these two movements supposes a helical contact tool-work piece generating the removal of the chip. In Figure 1, the initial chip section is shown for low values of feed in comparison with the depth of cut, *d*.

The size and form of the chip and the part of the material that remains without machining are, among others, function of the feed, *f*, the depth of cut, *d*, and the geometry and positioning of the cutting insert. The main position angle of the tool has a relevant influence on the shape of the chip, meanwhile the radius of the tool, *R*, is an important factor in the control of the surface rugosity of the machined part.

**Figure 2:**
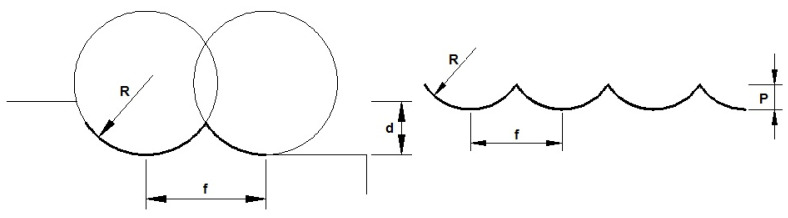
Theoretical surface profile on turning processes.

On turning operations, if the feed rate is much lower than the cutting speed, the profile generated by the tool on the surface of the machined part adopts the shape shown in Figure 2.

From a geometry point of view, it is demonstrated that the depth of cut has no influence on the final surface roughness profile, being feed and tool radius the most relevant factors on it. Some authors [23] have found out the mathematical theoretical expression for arithmetic average roughness, *R_a_,* shown in Equation (1):

(1) $$R_{a}=\frac{1}{f}\int_{0}^{f}\left|f\left(x\right)\right|dx=\frac{2}{f}\left(R^{2}\arcsin\frac{\sqrt{2kR-k^{2}}}{R}-\left(\frac{R^{2}}{f}\arcsin\frac{f}{2R}+\frac{1}{4}\sqrt{4R^{2}-f^{2}}\right)\sqrt{2kR-k^{2}}\right)$$

where $k=R-\frac{R^{2}}{f}\arcsin\frac{f}{2R}-\frac{1}{4}\sqrt{4R^{2}-f^{2}}.$

Nevertheless, it is demonstrated that *R_a_* can be approximated to Equation (2) with a high degree of accuracy for low values of feed [24].

(2) $$R_{a}≃\frac{f^{2}}{18\sqrt{3}R}$$

## 2. Experimental Details

### 2.1. Methodology for the Friction and Wear Analysis

With the objective of analysing the tribological behaviour between the cutting insert and the Cu-Ni 70/30 ASTM B122 alloy, friction tests were carried out in a pin-on-disk tribometer (Microtest, S.A., Madrid, Spain). Three lubrication conditions—dry, flood and LIL—were considered.

**Figure 3:**
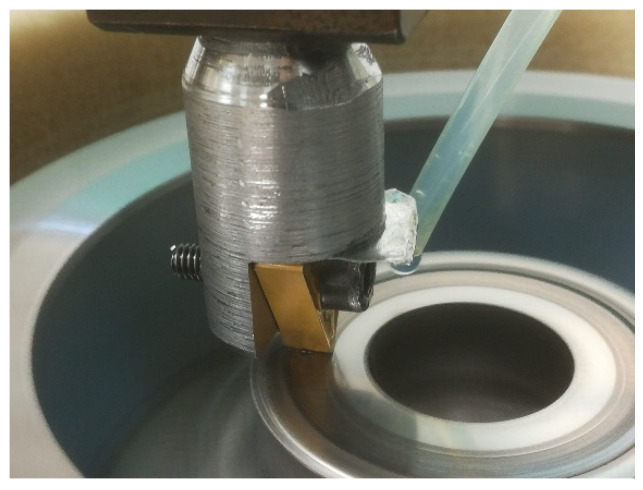
Detail of the contact between the surface of the workpiece and the tool tip.

TNMG 160408-MM2025 turning inserts (SANDVIK Corp., Stockholm, Sweden) were selected for this research. It is important to point out that they have been fixed vertically, ensuring the contact between the surface of the workpiece and the tool tip, as depicted in Figure 3. The cutting insert consisted of a 0.8-mm nose radius cemented carbide tool coated by TICN + Al_2_O_3_ + TIN CVD from Sandvik Coromant (SANDVIK Corp., Stockholm, Sweden). This tool was chosen because there are no specific cutting tools for machining Cu-Ni 70/30 and the insert is recommended for general purposes.

Tests were carried out with a linear speed of 45 m/min, which is within the range of turning processes. The normal force, *Fn,* was selected to be 20 N, under the three different lubrication conditions pointed out before. 20 N was the maximum load applicable in the tribometer for the studied sliding speed ensuring a homogeneous and stable contact in the sliding process.

Taking into account the dimensions of the insert and the width of the sliding track, the pressure in the contact evolved from several hundred MPa to 15 MPa, from the beginning to the end of a 1000-m test, respectively. Although at 1000 m the pressure is quite low, at the beginning of the test, the involved normal stress is similar to that reached in turning processes and reported by other authors [25]. Three different sliding distances, *L*, (1000, 2000 and 3000 m), were chosen to evaluate the tool wear. Thus, a total of nine experiments were carried out using a different new tool tip for each.

In the case of LIL lubrication, a drop of mineral cutting oil for general purposes from WD-40 manufacturer (WD-40 Company, Pine Brook, NJ, USA), with a viscosity 22–25 cSt, was supplied at the beginning of the process. Under flood condition, synthetic green coolant with a viscosity of 40 cSt was supplied, ensuring that the contact interface was always wet.

**Figure 4:**
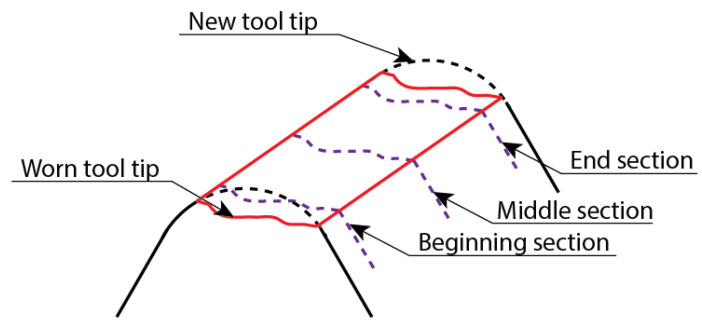
Schematic draw of new and worn tools and selected sections for wear analysis.

To measure the wear on the cutting insert tip, a three-dimensional profilometer FormTalysurf 50 (Taylor Hobson Inc., Leicester, UK) was employed. An amplitude of 1 mm and a resolution of 1 μm, were selected. Three 2D profiles of the tool tip, located at the beginning, in the middle and at the end of the insert breadth, were obtained at every insert, before and after the wear test was carried out (Figure 4). The beginning and end sections were separated 1 mm from the border to avoid the influence of the edges. From the overlapping of these 2D profiles, corresponding to the used and the unused insert, respectively, the mean-worn area was obtained.

The total volume of the material removed in the insert, *Vol*, was finally worked out multiplying the wear mean area by the thickness of the insert. The specific wear rates of the cutting tool under the three lubrication conditions experimented herein were obtained by dividing the volume of the material removed by the product of the normal force, *Fn*, and the sliding distance, *L*, as can be seen in Equation (3):

(3) $$K\left[\frac{mm^{3}}{N\cdot m}\right]=\frac{Vol}{Fn\cdot L}$$

It is important to explain that these tribological tests are not intended to replace the classical long cutting tests to evaluate tool wear until the end of the tool life. However, they can provide some interesting information about the friction phenomenon, the wear of the cutting tool and the interaction that takes place in the contact tool-workpiece under different lubrication conditions, since the evaluation of these concepts during the real cutting process becomes difficult.

### 2.2. Methodology for the Surface Integrity Analysis

Following the methodology established by the authors previously [1], the turning tests conditions of ASTM B122 alloy, under fixed cutting speed of 67.7 m/min, are summarized in Table 1. Short cutting tests consisting of turning an axial length of 30 mm, were developed for each cutting condition. The total length of the cylinder (130 mm) was divided according to each feed rate level. These parameters were established, according to previous trials, until a stable working range was found. For a depth of cut of 1 mm, feed rates are lower than for the other cases, see Table 1, due to the difficulty of turning this material under these challenging conditions. Besides, for a 1-mm depth of cut, feed rates higher than 0.1 mm/rev leads to a poor surface quality.

To carry out the experimental tests, a semi-automatic 10-kW lathe (Microcut H-2160, Buffalo Machinery Co., Ltd., Taichung, Taiwan) with a maximum spindle speed of 2000 rpm was used. TNMG 160408-MM2025 turning inserts and a MTJNR 2525M 16M1 tool holder from Sandvik Coromant were chosen (SANDVIK Corp., Stockholm, Sweden). For flood tests, a flow rate of 85 L/h of synthetic green coolant was selected. For low initial lubrication conditions tests a quantity of 2 mL was provided to carry out the turning of the whole cylinder. Taking into account the machining time employed under these conditions, the equivalent flow rate of oil was about 0.6 L/h. The lubrication step was repeated at the beginning of each trial, since the lubricated surface is removed in each previous operation. In the industrial field the lubrication step could be applied in the returning path of the tool, avoiding additional non-productive times. The maximum cutting speed was selected, according to the cylinder diameter, to avoid the loss of the cutting oil due to the existing centrifugal force.

To evaluate the surface roughness parameters, three-dimensional roughness tests were carried out by the FormTalysurf 50 profilometer (Taylor Hobson Inc., Leicester, UK). The dimension of the plotted area was 5 × 4 mm^2^. For that, one hundred travel paths were carried out in the direction of the width at a velocity of 1 mm/s, taking a total of 2000 points per length. The data were subsequently processed using the specific Talymap Gold software (6.0, Taylor Hobson Inc.). The surface shape was suitably rectified and removed by using a cylindrical filter. A 0.8 mm Gaussian filter was applied in all cases, separating the waviness component from the roughness profile.

With the objective of obtaining the arithmetic average roughness evolution for the turning process of Cu-Ni ASTM B122 alloy under LIL condition, the experiments evaluated are defined in Table 2.

Carrying out an ANOVA analysis of the three first depth of cut values, that is, 0.125, 0.25 and 0.50 mm, the most relevant factors in the variation of the surface roughness were obtained. Different regression models to predict the arithmetic average roughness evolution under LIL condition were adjusted by applying the least-squares method.

## 3. Experimental Results and Discussion

### 3.1. Pin on Disk Friction and Wear Results

**Figure 5:**
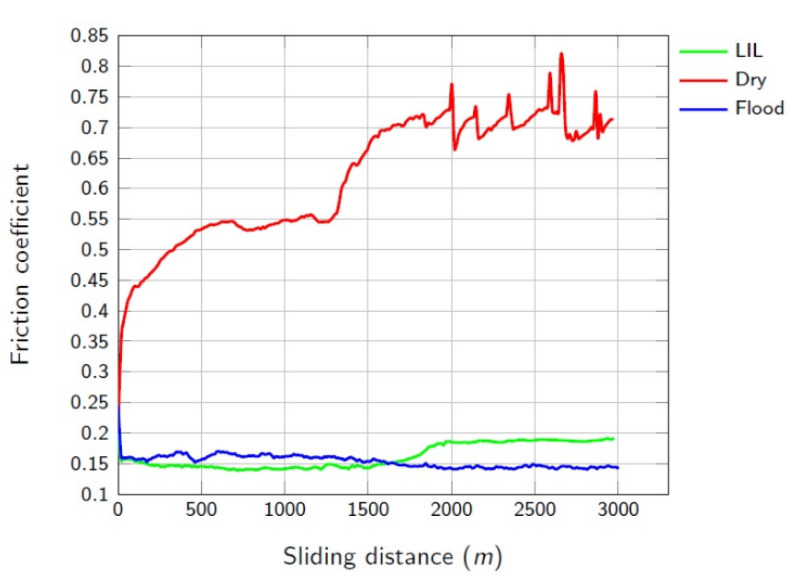
Friction coefficient evolution for 3000-m sliding distance pin-on-disk test.

Figure 5 shows the evolution of the coefficient of friction (COF), under the three different lubrication conditions considered in this research. As expected, COF under dry condition is extremely high in comparison with flood and LIL lubrication. At the same time, the evolution of COF versus the sliding distance shows an unstable and increasing behaviour, specially from 1700-m sliding length on. Clearly, the abrasive wear mechanism defines this tribological system. The rapid wear produced in the cutting tool generates hard particles that promote the abrasive behaviour of the contact. For a long sliding distance, the states of the worn surfaces provoke an irregular contact between the surfaces leading to a chaotic behavior of the COF value.

**Figure 6:**
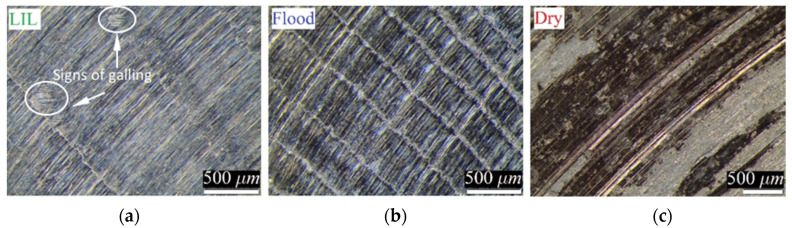
Cu-Ni wear tracks for different conditions: (**a**) LIL, (**b**) Flood and (**c**) Dry.

Under flood lubrication conditions, the evolution of the COF value is almost constant, and the contact between the tool and the Cu-Ni part is more regular with a typical value of 0.15, as it can be observed in Figure 5. Figure 6b shows the surface of the Cu-Ni part after 1000-m sliding distance. A great plastic deformation can be observed. Ploughing is characteristic and some dragged material is moved regularly in the sliding direction. This effect is limited by the instability of the accumulated material and then, the longitudinal material flow is interrupted and starts again. This phenomenon explains the regular transversal hills observed in the track.

In the case of LIL lubrication, initially, and up to 1700 m, the average friction coefficient obtained is 0.14, slightly lower than the one obtained under flood conditions. Nevertheless, after 1700 m of sliding distance, the friction coefficient grows up to an average value of 0.19. It means that the quantity of oil provided at the beginning of the test is enough to guarantee an effective lubrication until 1700 m. From this point on, the starvation of the lubricant promotes the rise of the COF value, what suggests the need of re-supplying more lubricant. However, even under lubricant starvation conditions, the COF value is relatively low and reasonable for machining applications and far from the typical values obtained under dry condition. The residual film of lubricant oil created on the part surface guarantees the typical boundary lubrication condition in the contact. Some evidence of micro-galling can be found at 1000-m sliding distance (Figure 6a). The small circled signed areas could be the result of small hard adhesions between the surfaces, similar to those found by other authors, for example, in soft coatings [26]. Nevertheless, it is demonstrated than with a very low quantity of cutting oil, provided at the beginning of the process, it is possible to maintain controlled and better lubrication conditions than for continuous flood lubrication. The best conditions of LIL lubrication are logically limited until the lack of lubricant, but the sliding friction is adequate even after.

**Figure 7:**
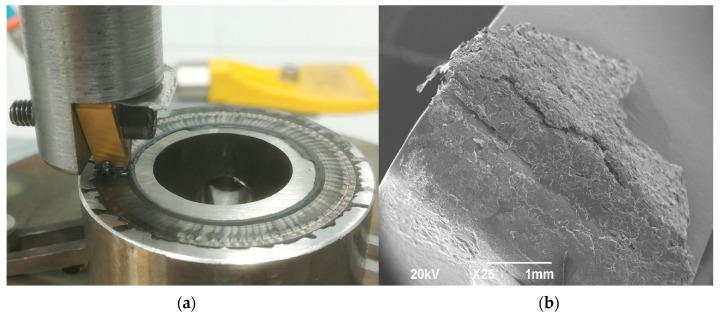
(**a**) General view of the Cu-Ni 70/30 alloy-tool friction system. (**b**) Detail of the grease layer formed by combination of the cutting oil and Cu-Ni particles.

**Figure 8:**
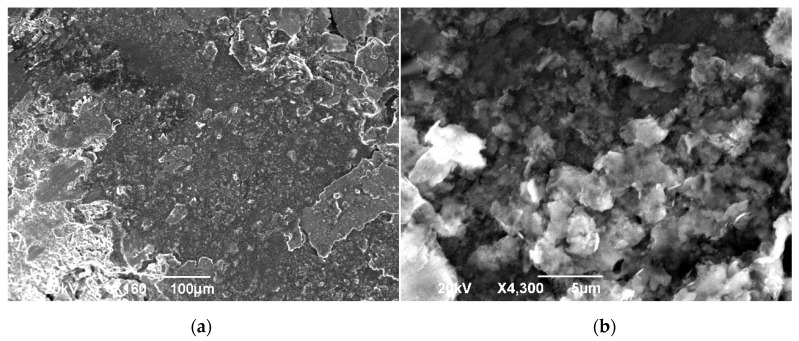
(**a**) SEM view of grease located in the tool tip. (**b**) Detail of Cu-Ni particles.

During the sliding under LIL conditions, a mixture of the debris created by wear and the oil used as lubricant is formed. This mixture seems to behave like a layer of grease, and it is an indicator that particles of debris are present in the contact area. According to the friction fundamentals, particles existing between the frictioned surfaces can modify the sliding conditions. If the particles trapped in the contact area are movable, they have a 3D abrasive effect, increasing the grooving and wear rate, but if particles are fixed, they can act forming a tribolayer [27], protecting the surface and diminishing the COF. This effect appears if the debris is present in a humid medium [28], providing effective lubrication in the contact zone (Figure 7). In Figure 8, it can be observed that the sizes of the Cu-Ni particles existing in the generated layer during the sliding evolution were within 1 to 3 μm. Nevertheless, some particles larger than 5 μm exist. The range of sizes observed contributes to maintaining the particles in the contact area as they can be considered small and active. Moreover, curve pins improve this effect, incorporating previously expelled particles into the track [29].

This explanation is compatible with the research of other authors who described the positive effect of solid lubricants with particle sizes in the range of 1 to 150 μm in terms of tool wear reduction and surface roughness. This fact has been repored for materials such as Inconel [30], Ti6Al4V [31] or different steels [32]. Particularly, Srivastava et al. [33] developed the turning process of Al 6061 alloy with the used of 2-μm solid lubricant particles, enhancing the surface roughness results, which is compatible with the results obtained herein.

**Figure 9:**
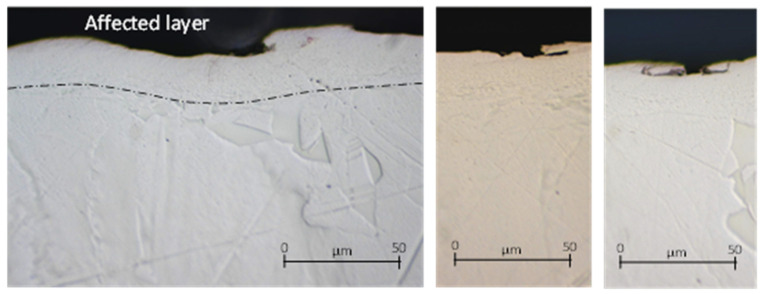
Micrographs of the part surface after a pin-on-disk test under dry condition: 1000-m test.

Regarding with the effect of friction on the surface, dry friction promotes many irregularities on the part surface (Figure 9), and some hints of material pulled out and even peeling were observed.

**Figure 10:**
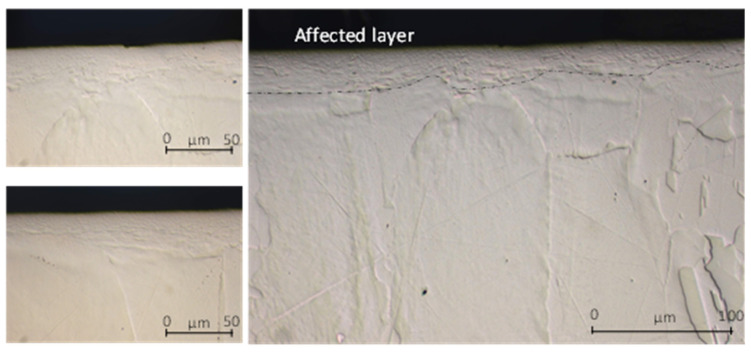
Micrographs of the part surface after a pin-on-disk test under LIL condition: 1000-m test.

**Figure 11:**
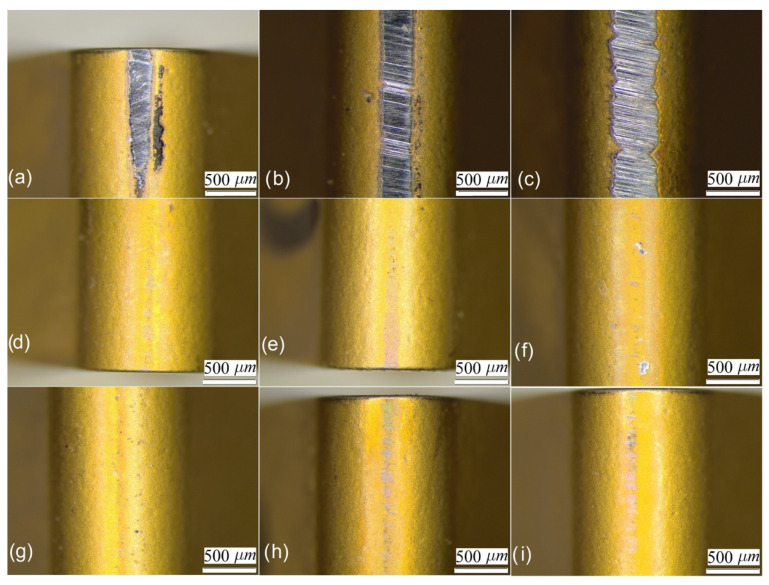
Tool wear evolution. Dry: (**a**) 1000 m, (**b**) 2000 m and (**c**) 3000 m, flood: (**d**) 1000 m, (**e**) 2000 m and (**f**) 3000 m, LIL: (**g**) 1000 m, (**h**) 2000 m and (**i**) 3000 m.

Otherwise, LIL lubrication allows smooth surfaces to be obtained, as can be seen in Figure 10, which shows that the sub-surface was affected by the friction process. Moreover, the thickness of the deformed layer was similar for both conditions, dry and LIL. As expected, without lubrication, the high COF value results in significant wear on the cutting insert. According to Figure 11, that shows the wear evolution at the tool tip for the three lubrication conditions, the tool wear under dry conditions is remarkable. Under dry conditions and after a sliding length test of 1000 m, the coating of the insert was removed, while under flood or LIL lubrication the coating remained through the 3000-m test.

**Figure 12:**
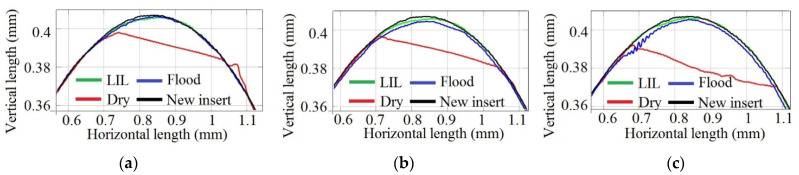
Profiles of a section of the cutting insert tip under different lubrication conditions for (**a**) 1000 m, (**b**) 2000 m and (**c**) 3000 m of sliding distance tests.

In Figure 12 the central section of the tool for sliding lengths of 1000, 2000 and 3000 m are shown under the three different lubrication conditions considered herein. The sections were obtained by 2D profilometry and compared with the corresponding values of a new tool.

Abrasion is the most remarkable wear phenomenon. According to Figure 11, adhesion wear is not noticeable, what might indicate a low chemical affinity between the coating of the insert and the Cu-Ni alloy. Nevertheless, the difference in the insert wear without lubrication compared with those under flood and LIL conditions is significant. Furthermore, it can be observed that for the 3000-m sliding tests, the tool wear under LIL condition is slightly lower than for flood lubrication. Effectively, the insert tested under flood lubrication shows more individual wear defects as the worn surface under LIL conditions is more uniform (Figure 9f,i, respectively).

A wear average area of the cutting insert was obtained, taking into account the different sections of the tool considered (Table 3). The wear volume was obtained by multiplying the average worn area by the thickness of the insert, that is 4.8 mm. A reduction of the tool wear of 87% is observed under LIL conditions in comparison with the non-lubricated test for 1000 m, whereas a reduction of almost 95% is observed for 3000 m.

A 73% wear reduction for LIL conditions respect to the flood lubrication for a distance length of 3000 m is observed as well. It is definitely demonstrated that the layer created by the mixture of oil and metal particles provides a more effective lubrication than the typical flood lubrication condition, where a great amount of the debris formed is thrown out of the sliding path tool-alloy.

**Figure 13:**
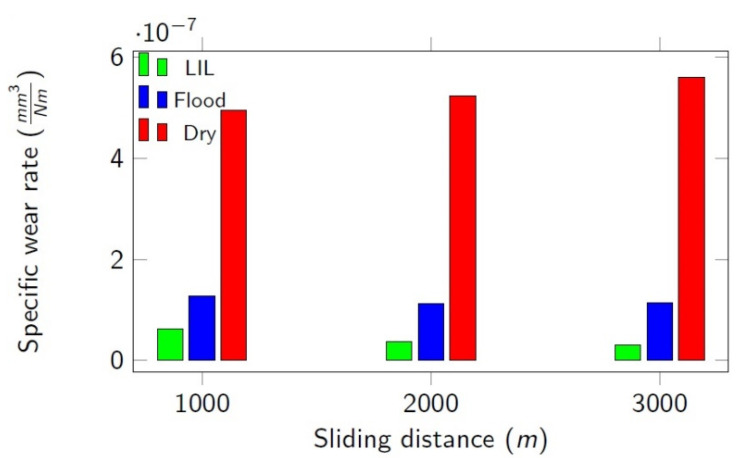
Specific wear rate, K [mm^3^/Nm].

By using Equation (3), the specific wear rate was obtained, which is represented in Figure 13. The lowest specific wear rate found, corresponding to LIL conditions, was $4.3\times 10^{-8}$ mm^3^/Nm. This result represents tool life improvements of around 63% and 92% if compared with flood lubrication and dry conditions, respectively ($1.18\times 10^{-7}$ and $5.26\times 10^{-7}$ mm^3^/Nm).

### 3.2. Surface Integrity Results in Turning Process

**Figure 14:**
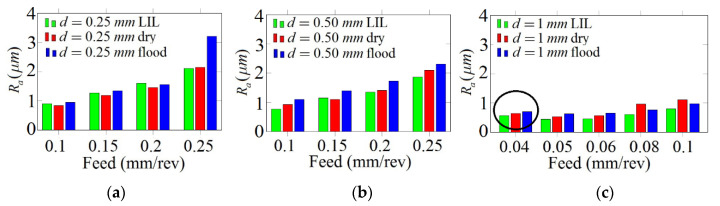
*R_a_* evolution for different depths of cut: (**a**) 0.25 mm, (**b**) 0.50 mm and (**c**) 1 mm.

Figure 14 shows the evolution of *R_a_* against the feed rate for a 67.7-m/min cutting speed for different depths of cut and lubrication states.

As was expected that the *R_a_*-value would increase directly with the feed rate under any lubrication condition, which agrees with the theoretical relationship between *R_a_* and the cutting parameters (Equation (2)). According to Figure 14c, for a 1-mm depth of cut, it can be highlighted that there is an odd result for 0.04-mm/rev feed rate. In this case, high vibrations were observed during machining, worsening the surface finish, what explains the anomalous initial tendency observed in the corresponding graph.

**Figure 15:**
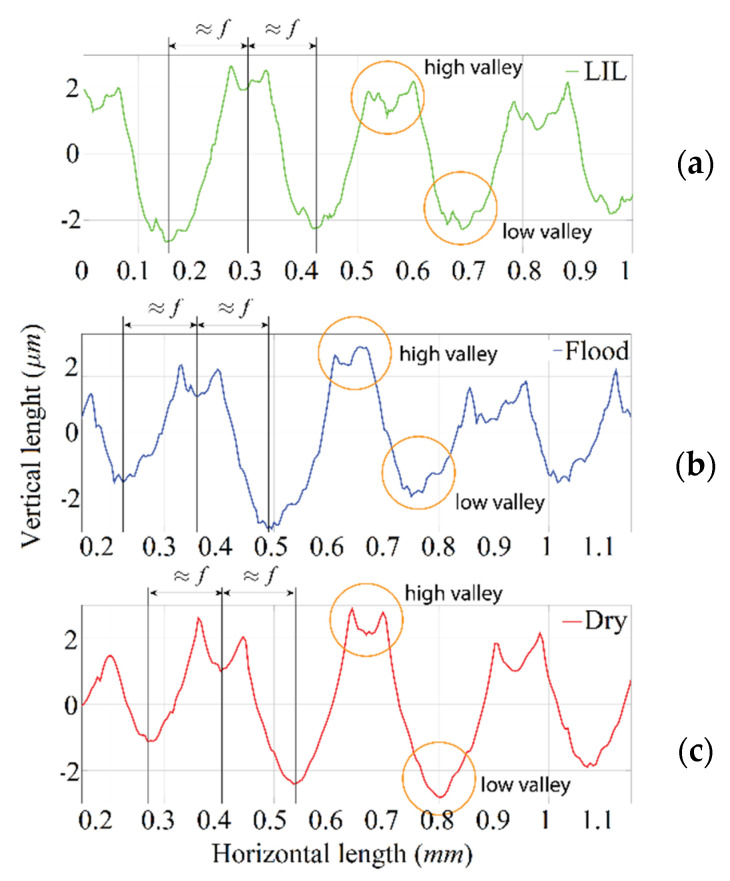
Roughness profiles for 0.25 mm depth of cut and 0.15 mm/rev of feed rate; (**a**) Turning under LIL condition; (**b**) Turning under flood lubrication; (**c**) Turning in dry condition.

Figure 14a, that corresponds to 0.25-mm depth of cut, depicts the lowest roughness values obtained under dry cutting. This result seems to be contrary to the expected one based on the previous pin-on-disk tribological analysis that predicted the lowest roughness values under LIL conditions. However, this behaviour could be explained by analysing the roughness profiles of the machined parts for the depth of cut considered (Figure 15).

When the feed rate is lower than 0.2 mm/rev, there is a bad interaction between the cutting tool and the workpiece, which does not result in a stable and homogeneous cutting process. During one revolution, the material is dragged, being cut at the next one. This oscillation in the cutting process generates an irregular roughness profile, producing high and low valleys separated by a distance that varies according to the feed rate applied. Thus, for all three of these lubrication cases, below the critical 0.20-mm/rev feed rate, the roughness is not produced naturally and turns out to be higher than expected, especially for the lowest cut depth (see Figure 15). On the other hand, above 0.20 mm/rev, the experimental *R_a_*-values, that are lower than the theoretical ones, are always the best under LIL conditions (Figure 14b,c). The disagreement between the experimental and theoretical *R_a_*-values can be explained according to the plastic deformation phenomenon experimented by the material during the turning operation. Definitely, better results are obtained for LIL than for flood lubrication with much less lubricant.

**Figure 16:**
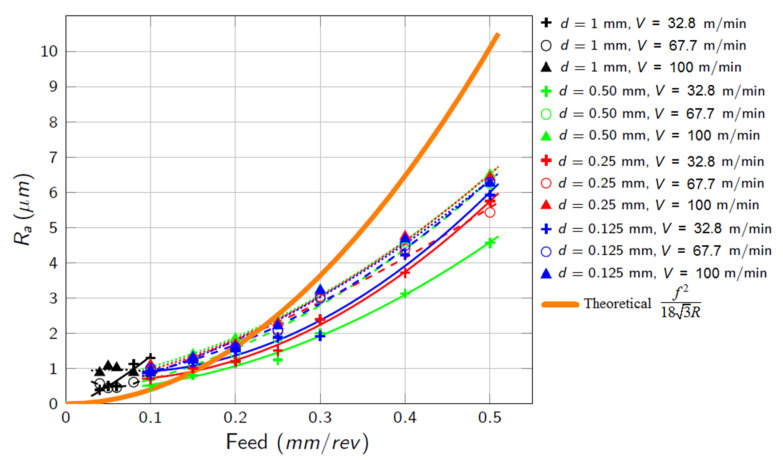
*R_a_* evolution under LIL condition tests. V is the cutting speed and d is the depth of cut.

This can be explained taking into consideration the higher plastic deformation produced in Cu-Ni material under flood conditions compared to LIL lubrication, as observed in the pin-on-disk friction tests. For some cutting conditions at 0.25 mm/rev feed rate, *R_a_*-values for dry machining are even lower than in flood lubrication, which can be attributed to an electrochemical interaction between the cutting insert and the workpiece, enhanced by the presence of the coolant [34]. As a summary of the results, Figure 16 shows the evolution of *R_a_* under LIL test conditions for the range of the cutting parameters experimented.

It can be seen that the feed rate is the most relevant influence factor for the *R_a_*. In almost all cases, the experimental values are adjusted to a second-degree polynomial model as a function of the feed rate. Only the results corresponding to 1-mm depth of cut do not fit this model due to the low differences between the feed rates analysed.

The experimental values definitely do not agree with the expected theoretical behaviour. While theoretically roughness is a function of feed and tool radius, variations in cutting speed and depth of cut have an influence on it too. Besides, in machining operations the concept of surface roughness must be extended to the concept of surface integrity, which includes the phenomena of vibration, plastic deformation of the material and interaction and contact between the cutting tool and the machined part, among other factors. It has been reported by some authors that the surface roughness values obtained experimentally can be greater or lower than the ones calculated analytically. Ductile materials with a high degree of work-hardening generally show improved surface roughness, while brittle materials usually give worse results [23]. Besides, the removed material and its deformation during machining may cause a ploughing effect [35], distancing the surface roughness levels from the theoretical ones.

The influence of each input factor on surface roughness has been studied by ANOVA analysis (Table 4). It is demonstrated that the influence of the depth of cut on *R_a_* is low and only relevant for high feed rates and low cutting speeds, resulting in an optimum behaviour for a 0.50-mm cut depth. Although the increment of the cutting speed makes the surface roughness increase (up to 44% for the highest feed rate), the relevance of the depth of cut on surface roughness for 100 m/min turning tests is negligible.

Taking into consideration the previous analysis, some empirical models are proposed to predict the evolution of *R_a_* under LIL conditions (Table 5).

The last model in Table 5 has been traditionally used to predict surface roughness r turning operations. It can be summarized that the consideration of the depth of cut, *d*, in the model function does not implies significant improvements. Thus, models 2 and 5 are equally consistent and models 6 and 7 are even better. Eventually, although the feed rate, *f*, is the most important variable according to our ANOVA analysis, it is essential to include the cutting speed, *V*, in the correlation functions.

## 4. Conclusions

In this paper, the tribological behaviour of the Cu-Ni ASTM B122 alloy and TNMG 160408-MM2025 turning insert system has been analysed under different lubrication conditions. Moreover, a new lubrication approach named low initial lubrication (LIL) and based on application of a low quantity of lubricant at the beginning of the process has been proposed. The surface roughness of the machined parts has been characterized and the most relevant parameters affecting the *R_a_* of the workpiece surface have been pointed out and the wear phenomena involved have been studied. According to the results, the following conclusions are obtained:

- COF under dry conditions is extremely high, which can lead to a higher cutting force and great temperature during dry machining. By applying continuous flood lubrication, the COF is significantly reduced.
- Under LIL conditions, the COF is similar to the value obtained under flood lubrication conditions. For that purpose, the application of only one droplet of lubricant is necessary. This beneficial effect can be attributed to a layer of lubricant created by the mixture of oil and the debris during friction. Although this effect is limited, the effectiveness of this lubrication system has been demonstrated for long run distances.
- Tool wear is considerably reduced under LIL conditions, implying an increase in tool life.
- The surface integrity analysis for the machined parts has been performed based on the *R_a_* parameter. It has been verified that the roughness is always lower under LIL conditions, what can be explained in comparison to flood traditional lubrication due to the lower plastic deformation of Cu-Ni alloy during the cutting process.
- By using ANOVA analysis, the most relevant parameters affecting *R_a_* under LIL conditions have been obtained. The feed rate is the most important factor, with a contribution of 95%. The cutting speed, although is less relevant, should also be taken into consideration. Alternative empirical models have been proposed to predict the surface roughness under LIL condition for the turning process of Cu-Ni ASTM B122 alloy.

## Figures and Tables

**Table 1:** Combination of cutting parameters for the experimental tests.

Depth of Cut (mm)	Feed Rate (mm/rev)	Lubrication Condition
0.25	0.1, 0.15, 0.2, 0.25	Dry, flood, LIL
0.50	0.1, 0.15, 0.2, 0.25	Dry, flood, LIL
1	0.04, 0.05, 0.06, 0.08, 0.1	Dry, flood, LIL

**Table 2:** Combination of cutting parameters for the cutting tests carried out under LIL conditions.

Depth of Cut (mm)	Feed Rate (mm/rev)	Cutting Speed (m/min)
0.125	0.1, 0.15, 0.2, 0.3, 0.4, 0.5	32.8, 67.7, 100
0.25	0.1, 0.15, 0.2, 0.3, 0.4, 0.5	32.8, 67.7, 100
0.50	0.1, 0.15, 0.2, 0.3, 0.4, 0.5	32.8, 67.7, 100
1	0.04, 0.05, 0.06, 0.08, 0.1	32.8, 67.7, 100

**Table 3:** Average wear area (A) and wear volume (Vol) in the insert in pin-on-disk experiments of the pair Cu-Ni alloy-tool insert.

Length	Wear	LIL	Flood	Dry
1000 m	A (μm^2^)	260.59	532.91	2079.67
	Vol (mm^3^)	0.00124	0.00254	0.00991
2000 m	A (μm^2^)	308.68	944.22	4388.49
	Vol (mm^3^)	0.00147	0.00450	0.02090
3000 m	A (μm^2^)	382.43	1439.76	7047.31
	Vol (mm^3^)	0.00182	0.00686	0.03357

**Table 4:** ANOVA analysis results for arithmetic average roughness under LIL conditions.

Factor	DF	Adj. SS	Adj. MS	F	P	Contribution (%)
d	2	0.376	0.188	3.47	0.047	0.19
f	6	185.218	30.8696	570.24	0	95.13
V	2	5.392	2.6962	49.81	0	2.77
f·V	12	1.234	0.1028	1.9	0.088	0.63
d·V	4	0.922	0.2306	4.26	0.01	0.47
d·f	12	0.26	0.0217	0.4	0.949	0.13
ANOVA Error	24	1.299	0.0541	-	-	0.67
Total	62	194.702	-	-	-	100

**Table 5:** Predictive models for arithmetic average roughness under LIL conditions. *V* (mm/s), *f* (mm/rev) and *d* (mm). SSE is the sum of squared error.

Model	Parameters	SSE (μm^2^)	Standard Deviation (μm)
$R_{a}=a\cdot f^{b}$ [1]	a = 15.9839 b = 1.4415	10.90	0.42
$R_{a}=a\cdot f^{b}\cdot V^{c}$ [2]	a = 3.5730 b = 1.4321 c = 0.2149	5.26	0.29
$R_{a}=a+f^{b}\cdot V^{c}$ [3]	a = 0.5564 b = 1.7426 c = 0.4129	7.03	0.34
$R_{a}=a+b\cdot f+c\cdot V$ [4]	a = −1.5838 b = 12.9461 c = 0.0006	9.72	0.39
$R_{a}=(a+b\cdot V)\cdot f^{c}$ [5]	a = 12.1269 b = 0.0038 c = 1.4324	5.49	0.29
$R_{a}=a\cdot f^{b}\cdot V^{c}+e$ [6]	a = 3.5262 b = 1.7072 c = 0.2354 e = 0.4266	4.59	0.27
$R_{a}=(a\cdot f^{2}+b\cdot f+c)\cdot v^{e}$ [7]	a = 3.9542 b = 0.5612 c = 0.0911 e = 0.2137	4.36	0.26
$R_{a}=a\cdot f^{b}\cdot V^{c}\cdot d^{e}$ [8]	a = 3.4343 b = 1.4321 c = 0.2126 e = −0.0399	4.93	0.28

## Nomenclature

*f*	feed rate (mm/rev)
*d*	depth of cut (mm)
*V*	cutting speed (m/min)
*R*	tool radius
*R_a_*	arithmetic average roughness (µm)
*COF*	coefficient of friction
*Fn*	normal force (N)
*L*	sliding distance (m)
*Vol*	average wear volume (mm^3^)
*A*	average wear area (mm^2^)
*K*	specific wear rate (mm^3^/Nm)
